# A Dendritic Neuron Model with Adaptive Synapses Trained by Differential Evolution Algorithm

**DOI:** 10.1155/2020/2710561

**Published:** 2020-01-17

**Authors:** Zhe Wang, Shangce Gao, Jiaxin Wang, Haichuan Yang, Yuki Todo

**Affiliations:** ^1^Faculty of Engineering, University of Toyama, Toyama-Shi 930-8555, Japan; ^2^School of Electrical and Computer Engineering, Kanazawa University, Kanazawa-Shi 920-1192, Japan

## Abstract

A dendritic neuron model with adaptive synapses (DMASs) based on differential evolution (DE) algorithm training is proposed. According to the signal transmission order, a DNM can be divided into four parts: the synaptic layer, dendritic layer, membrane layer, and somatic cell layer. It can be converted to a logic circuit that is easily implemented on hardware by removing useless synapses and dendrites after training. This logic circuit can be designed to solve complex nonlinear problems using only four basic logical devices: comparators, AND (conjunction), OR (disjunction), and NOT (negation). To obtain a faster and better solution, we adopt the most popular DE for DMAS training. We have chosen five classification datasets from the UCI Machine Learning Repository for an experiment. We analyze and discuss the experimental results in terms of the correct rate, convergence rate, ROC curve, and the cross-validation and then compare the results with a dendritic neuron model trained by the backpropagation algorithm (BP-DNM) and a neural network trained by the backpropagation algorithm (BPNN). The analysis results show that the DE-DMAS shows better performance in all aspects.

## 1. Introduction

The human brain consists of billions of neurons, and a single neuron cell is constituted by a cell body, an axon, a cell membrane, and a dendrite. Dendrites occupy more than 90 percent of the nerve cell organization and have a pivotal role in a human's learning process. The first artificial neuron was originally proposed by MuCulloch and Pitts in 1943 [1]. This model is an abstract and simplified model that was constructed according to the structure and working principle of a biological neuron membrane based on mathematics and algorithms called threshold logic.

The perceptron is a method for pattern recognition, which was first created by Rosenblatt in 1958 [2, 3]. It was the first artificial neural network model, laying the foundation for the neural network model. However, in Minsky Papert's analysis of Rosenbatt's single-layer perceptron from a mathematical perspective [4], the artificial neural network was criticized with an example of the XOR operation. The problem of how an intelligent system independently learns from an environment is not well solved, and the development of artificial neural networks (ANNs) has deteriorated. In the mid-1980s, scholars began to explore the inner logic of knowledge discovery in depth and discovered that inductive logic, especially incomplete induction logic, is a reasonable way to discover knowledge. Rumelhart et al. surprisingly discovered that the backpropagation error (BP) [5], which was invented by Werbos more than 10 years ago, can effectively solve the learning problems of hidden nodes in multilayer networks. It is not correct to accept Minsky's assertion that there may be no effective learning methods for multilayer networks. Since then, people's enthusiasm for ANN research has been rekindled.

However, researchers have argued that the use of McCulloch and Pitts's neuron is inadvisable because it disregards the dendritic structure in a real biology neuron. Koch and Segev [6, 7] proposed that the interaction between synapses and the action at the turning point of a branch can be approximated as logic operation. In recent years, several dendritic computing models considering the functions of dendrites in a neuron have been proposed in the literature. A dendritic morphological neural network (DMNN) which is based on the traditional morphological neural networks [8, 9] is proposed for solving classification problems [10] and 3D object recognition tasks [11]. A nonlinear dendritic neuron model equipped with binary synapses [11] is demonstrated to be capable of learning temporal features of spike input patterns. Most recently, a dendritic neuron model (DNM) with nonlinear synapses has been proposed [12–14]. Different from DMNN, DNM only considers a single neuron rather than the network of a couple of neurons and has shown great information processing capacity [15–19]. The DNM uses a pruning technique derived from an interesting biological phenomenon: in the early stages of neuron triggering, the selective removal of unnecessary synapses and dendrites does not cause neuron cell death [20, 21]. The DNM subtly solves nonlinear problems that cannot be well handled by the Koch model [22, 23]. The DNM has four layers in its structure. The input signal is triggered in the synaptic layer and then sequentially received by the dendritic layer. The membrane layer collects the output from each branch of the dendritic layer and sends the results to the somatic cell layer. By the pruning function of the DNM, the precise dendritic structure and morphology are simplified. After training, all mature neurons are approximately replaced by a logic circuit that consists of comparators, AND gates, OR gates, and NOT gates.

In this study, we use a dendritic neuron model with adaptive synapses (DMASs). Recent advances in neurobiology have highlighted the importance of dendritic calculation. In 2019, Beaulieu-Laroche and his team [24] discovered that dendrites are always active when the cell body of a neuron is active, which implies that the dendritic synapse has a role in the neural computing process. Based on this biophysical hypothesis, we develop a synaptic adaptable neuron network without parameters that need to be artificially adjusted. All synaptic layer parameters will be trained by the learning algorithm. The effectiveness of adaptive synapses will be proved in Section 4.3. Thus, we have to consider additional aspects in the choice of learning algorithms.

With the emergence of various new optimization algorithms, how to train an ANN has been discussed [25]. BP is very effective as an ANN training algorithm and can solve some nonlinear problems [5]. However, BP has certain limitations; for example, falling into a local minimum is easy, the convergence speed is slow, and it is prone to overfitting [26]. Differential evolution (DE) has been employed to train DMAS in our research. DE was first proposed by Storn and Price in 1997 [27]. It is a biological-inspired, population-based global optimization algorithm. Due to its simple concept, easy implementation, fast convergence, and excellent robustness, it has been more extensively utilized than other mainstream evolutionary algorithms, such as the genetic algorithm (GA) [28, 29], the evolutionary strategy (ES) [30, 31], and particle swarm optimization (PSO) [32] in recent years. DE is similar to the GA and ES but differs from them because a unique differential evolution operator is referenced in DE. DE has proven to be superior to many algorithms [33–35]. Because of these characteristics and the advantages of DE, it has been recognized by scholars in the field of ANNs [36, 37]. Also, DE has been applied in dendrite morphological neural networks [38].

Five realistic classifications problems are considered in our research to validate our model (DE-DMAS) : iris, BUPA liver disorders, breast cancer, glass, and Australian credit approval (ACA). All the datasets are preprocessed as the binary-classification problem. These five datasets have undergone preprocessing, including outlier repair to fill in missing values. We compare the experimental results of DE-DMAS, BP-DNM, and BPNN for these five datasets. Experimental results show that DMAS outperforms its peers in terms of test accuracy, sensitivity, specificity, receiver operating characteristic (ROC), and cross-validation.

The remainder of this paper is organized as follows: Section 2 introduces the structure of our model (DMAS). The learning algorithm (DE) is explained in Section 3. The experimental method is designed in Section 4.  Section 5 presents the analysis and discussion of the experimental results. The conclusions are provided in Section 6.

## 2. Dendritic Neuron Model with Adaptive Synapses

DMAS is applied in our research. The neuron model includes four layers: the adaptive synaptic layer, dendritic layer, membrane layer, and somatic cell layer. In this section, we detail the structure and principle of these four layers.

### 2.1. Adaptive Synaptic Layer

The synaptic layer receives and computes the input signal and sends the calculated results to the dendritic layer. Once the input signal exceeds the threshold, synapses will be fired. To simulate this process, we design a synaptic layer with a sigmoid faction as in the following equation:

(1) $$\begin{array}{c} Y_{i,m}=\frac{1}{1+e^{-k\left(w_{im}x_{i}-q_{im}\right)}}, \end{array}$$

where *x*_*i*_ is the input and *Y*_*i*,*m*_ is the output of the *m*-th (*m*=1,2,3,…, *M*) branch of dendrites. The *i* in *i*=1,2,3,…, *I* represents the number of inputs that have been normalized into [0, 1] from the dataset. *I* also represents the number of synapses on each dendrite. *k* is a tunable parameter which denotes the connection strength between presynaptic and postsynaptic neurons. To reduce the parameters that need to be adjusted in our study, *k* will be used as the training object. Due to the nature of the sigmoid function, this step has a minimal effect on the function. *w*_*im*_ and *q*_*im*_ are objects that also need to be trained by the learning algorithm; their values will be set initially within [−2, 2]. Because the synaptic layer works with these three training objects and inputs and no artificial adjustment parameters are needed, this synapse has an adaptive function [39]. The threshold *θ*_*im*_ is an important indicator for synapses and is calculated by the following equation:

(2) $$\begin{array}{c} \theta_{im}=\frac{q_{im}}{w_{im}}. \end{array}$$

**Figure 1:**
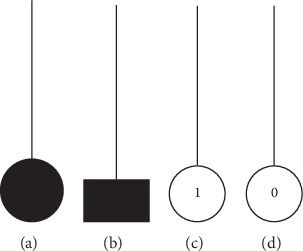
Four connecting states: (a) direct-connecting state; (b) opposite-connecting state; (c) constant-1 state; (d) constant-0 state.

After the synapse has been activated by the sigmoid function, it can adopt one of the 4 different states according to different ranges of *w*_*im*_ and *q*_*im*_. These states are described as the direct-connecting state (●), opposite-connecting state (^▂^), constant-1 state (①), and constant-0 state (⓪), as shown in Figure 1. According to the change of the values of *w*_*im*_ and *q*_*im*_, the four states are divided into the following six cases.

**Figure 2:**
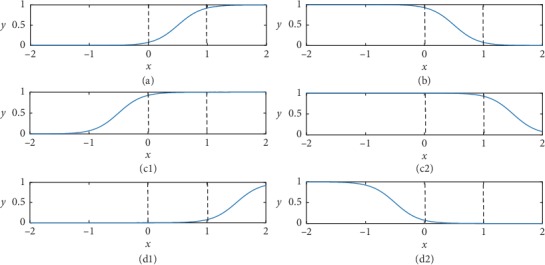
The synapse function figures for the four states: (a) direct-connecting state; (b) opposite-connecting state; (c1) constant-1 state; (c2) constant-1 state; (d1) constant-0 state; (d2) constant-0 state.

Case (a): direct-connecting state, when *w*_*im*_ > *q*_*im*_ > 0. In this state, if the value of the input *x*_*im*_ is greater than *θ*_*im*_, the value of the output approximately equals 1; otherwise, it equals 0. For example, when *w*_*im*_=1.0 and *q*_*im*_=0.5, the function can be shown in Figure 2(a), where the *X*-axis represents the value of the input *x* and the *Y*-axis represents the value of the output. Since the range of input is [0, 1], we only need to pay attention to the area between the two dashed lines.

Case (b): opposite-connecting state, e.g., when 0 > *q*_*im*_ > *w*_*im*_. In this state, if the value of the input *x*_*im*_ is less than *θ*_*im*_, the value of the output approximately equals 1; otherwise, it equals 0. A synapse in this state works as a logic NOT operation. For example, when *w*_*im*_=−1.0 and *q*_*im*_=−0.5, the function diagram is as shown in Figure 2(b).

Case (c1): constant-1 state when *w*_*im*_ > 0 > *q*_*im*_. For example, when *w*_*im*_=1.0 and *q*_*im*_=−0.5, the function diagrams are as shown in Figure 2(c1).

Case (c2): constant-1 state when 0 > *w*_*im*_ > *q*_*im*_. For example, when *w*_*im*_=−1.0 and *q*_*im*_=−1.5, the function diagrams are as shown in Figure 2(c2). In cases (c1) and (c2), regardless of the value of the input *x*_*i*,*m*_, the output remains 1.

Case (d1): constant-0 state when *q*_*im*_ > *w*_*im*_ > 0. For example, when *w*_*im*_=1.0 and *q*_*im*_=1.5, the function diagrams are as shown in Figure 2(d1).

Case (d2): constant-0 when state *q*_*im*_ > 0 > *w*_*im*_. For example, when *w*_*im*_=−1.0 and *q*_*im*_=0.5, the function diagrams are as shown in Figure 2(d2). In cases (d1) and (d2), regardless of the value of the input *x*_*im*_, the output remains 0.

### 2.2. Dendritic Layer

The outputs of the synaptic layer are calculated by the dendritic layer using multiplication. Because the sigmoid function is employed, the outputs are approximately equal to either 1 or 0. The outputs of the dendritic layer are also approximately equal to either 1 or 0. The dendrites work the same as a logic AND operation. The equation is

(3) $$\begin{array}{c} Z_{m}=\overset{I}{\underset{i=1}{\prod }}Y_{im}. \end{array}$$

### 2.3. Membrane Layer

The membrane accepts the output of the dendritic layer as the input and linearly sums the values. The summation can be approximately simulated with logic OR operations. The equation is

(4) $$\begin{array}{c} V=\overset{M}{\underset{m=1}{\sum }}Z_{m}. \end{array}$$

### 2.4. Somatic Cell Layer

The somatic cell layer will receive the signal from the membrane. The signal is calculated using the sigmoid function as follows:

(5) $$\begin{array}{c} O=\frac{1}{1+e^{-k_{\text{soma}}\left.\left(V-\theta_{\text{soma}}\right)\right)}}, \end{array}$$

where *k*_soma_ and *θ*_soma_ are set to 10 and 0.5, which were suggested to be the most promising setting in our previous papers [40, 41].

### 2.5. Simplified Model

**Figure 3:**
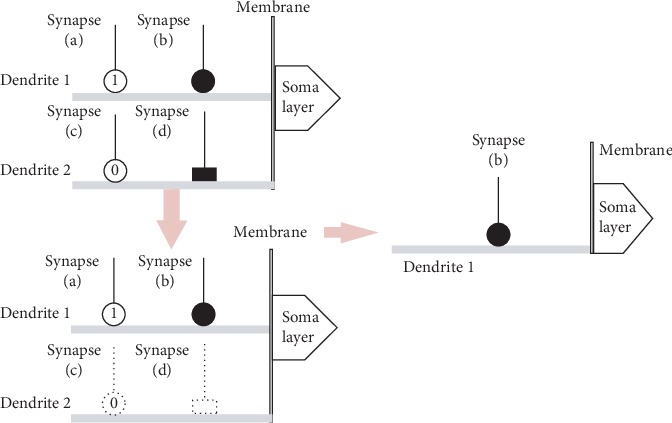
Simplified model.

We pruned the synapses and dendrites to obtain our simplified model. The synapse receiving the input signal is activated and converted into the constant-1, constant-0, direct-connecting, or opposite-connecting state. The activated signal is transmitted to the dendrites. These signals are multiplied in the dendrites, enter the membrane, and are received by the soma. When a synapse is converted into the constant-1 state, we will remove this synapse since 1 multiplied by any number is equal to the number itself. When a synapse on a dendrite is converted to the constant-0 state, we will remove this dendrite since 0 multiplied by any number equals 0. An example is shown in Figure 3. In the upper left diagram, dendrite (2) has a constant-0 state synapse (c) and an opposite-connecting state synapse (d). Because synapse (c) is in the constant-0 state, dendrite (2) is removed, including the other synapses, as shown in the lower left diagram. We refer to this step as dendrite pruning. In the lower left diagram, a constant-1 state synapse (a) and a direct-connecting state synapse (b) exist on dendrite (1). Since synapse (a) is in the constant-1 state, this synapse is removed. We refer to this step as synapse pruning. The diagram on the right shows the simplified model after pruning, and only dendrite (1) and synapse (b) remain.

## 3. Learning Algorithm

As previously mentioned, we use the three parameters of the synaptic layer as training objects. This space is a vast search space. We use the DE algorithm as the learning algorithm of DMAS. Since DE is excellent in global optimization [42], it can find the optimal solution faster in the immense search space.

DE demonstrates a fixed number of vectors that are randomly initialized in the search space. The new vectors evolve over time to explore the minimum of the objective function. In the process of evolution, arithmetical operators are combined with the operators of mutation, hybridization, and selection. A randomly generated starting population will be evolved to an optimal solution. DE has numerous strategies [43], and we used DE/rand/1/bin in this study. Other strategies include DE/best/1/exp and DE/rand/2/exp, and the preliminary experimental results had suggested that they were slightly inferior than DE/rand/1/bin because it has the simplest structure [44, 45]. Next, we will explain how DE works.

*Step 1*. Parameter setup. Select the population size *P* and restrict the boundary. Confirm the cross-probability CR, the impact factor *F* [46, 47], and the termination criterion of the maximum number of generations (*G*).
*Step 2.* Initialization of the population. Set the generation *g*=0. Initialize a population containing *P* individuals. The attributes of each individual include weights *w*, thresholds *q* and a *k*, described as *D*. The number of weights *w* and thresholds *q* equals the number of hidden layers (*M*) multiplied by the number of inputs (*I*) provided by the dataset. Thus, the population is considered to be a vector matrix of *P* rows and *D*(*D*=2 × *M* × *I*+1) columns. Each value of the weights *w* and thresholds *q* is initialized as a random real number in the range [−2, 2]. The value of the *k* is randomly initialized in the range [1, 10]. The following equation shows the content of the population:
  (6) $$\begin{array}{c} \left\{\begin{array}{c} x_{p}⟶\left(x_{1},x_{2},x_{3},\ldots ,x_{P}\right),\\ x_{p}=\left\{w_{\left(1\times 1\right)p},q_{\left(1\times 1\right)p},\ldots ,w_{\left(1\times M\right)p},q_{\left(1\times M\right)p},\ldots ,w_{\left(I\times M\right)p},q_{\left(I\times M\right)p},k_{p}\right\}. \end{array}\right. \end{array}$$

 
*Step 3*. Evaluation of the population. DE can be employed as a training algorithm for DE-DMAS. DE-DMAS can also be regarded as the evaluation function of DE. Therefore, the evaluation becomes a calculation of the mean square error (MSE), which will be formally defined in equation (12). In the experiment, the MSE of DMAS is the fitness at each step. Each up-to-date generation will be evaluated after the next mutation, crossover, and selection operations. In our research, the maximum number of generations is set to 1000. Thus, the evaluation function will be run 1001 times.
 
*Step 4*. Mutation operation. The mutation operation produces a mutation operator (*v*_*i*,*g*_). The process of production is shown in the following equation:

(7) $$\begin{array}{c} v_{i,g}=x_{r_{1},g}+F\left(x_{r_{2},g}-x_{r_{3},g}\right), \end{array}$$

  where *x*_*r*_1_,*g*_, *x*_*r*_2_,*g*_, and *x*_*r*_3_,*g*_ are randomly chosen from the population of this generation. If all individuals are regarded as points in the search space, then the mutation operation can be interpreted as follows: *v*_*i*,*g*_ is a new point after *x*_*r*_1_,*g*_ moves in the direction of *x*_*r*_3_,*g*_ to *x*_*r*_2_,*g*_ by *F* times the Euclidean distance between *x*_*r*_2_,*g*_ and *x*_*r*_3_,*g*_.
 
*Step 5*. Crossover operation. The crossover operation combines the mutation operator with the target individual, resulting in a new individual. DE involves two methods of crossover: binomial crossover and exponential crossover. Zaharie analyzed the performance of binomial crossover and exponential crossover [48] and suggested that exponential crossover is more affected by population size than binomial crossover. Binomial crossover is applied in our research. The following equation shows the crossover function:

(8) $$\begin{array}{c} u_{j,i,g}=\left\{\begin{array}{cc} v_{j,i,g,} & \text{if }{\text{rand}}_{j}\leq \text{CR or }j=j_{\text{rand}},\\ x_{j,i,g}, & \text{otherwise}. \end{array}\right. \end{array}$$

  Generate the random number rand_*j*_∈ [0, 1] for each dimension of each individual. If rand_*j*_ is less than CR in one dimension, then the target individual *x*_*i*,*g*_ is replaced by the mutation operator *v*_*i*,*g*_ in this dimension; otherwise, it remains the same as the target individual *x*_*i*,*g*_. Before this step, to ensure that the target individual hybridizes in at least in one dimension, a random integer *j*_rand_∈{1,2,3,…, *D*} is generated. When *j*=*j*_rand_, the target individual must hybridize in the *j*-th dimension.
 
*Step 6*. Selection operation. DE employs the mutation operator and the crossover operator to generate a son population and applies a one-to-one selection to compare the son individuals with the corresponding parent individuals. The better individuals are saved to the next-generation population. In DE-DMAS, the one-to-one selection operation can be described as follows:

(9) $$\begin{array}{c} x_{i,g+1}=\left\{\begin{array}{cc} u_{i,g}, & \text{if }{\text{MSE}}_{u_{i}}\leq {\text{MSE}}_{x_{i}},\\ x_{i,g}, & \text{otherwise}. \end{array}\right. \end{array}$$

Since DE employs a one-to-one selection method, the algorithm can ensure that the elitism will not be lost during the evolution process. In addition, one-to-one selection operation has a better ability to maintain population diversity than sequencing or competitive bidding selection [44]. The following Algorithm 1 summarizes the above steps, where two functions rnd_int and rnd_real return random integer and real numbers in the specified range, respectively.

## 4. Experimental Design

To achieve the best performance of the proposed method, it is first necessary to confirm the parameters. DE-DMAS has six main parameters. The parameters can be divided into fixed parameters and adjustable parameters. The best adjustable parameters are determined using the Taguchi method for each dataset [49], which is detailed in Section 4.2. In Section 4.3, we will prove the adaptability of synapses as mentioned above. Finally, DE-DMAS is compared with BP-DNM and BPNN, which are introduced in Sections 4.4 and Section 4.5, respectively. The five datasets adopted in our research are introduced in the following sections.

### 4.1. Dataset

The five datasets, which are obtained from UCI, are extensively applied in artificial intelligence research. The datasets have been standardized by maximum minimization to [0, 1] in our research. Their detailed introduction and summary are provided in Table 1.

The iris data were provided by Fisher in July 1988 [50–52]. The data have three classes: Iris Setosa, Iris Versicolour, and Iris Virginica. Each class has 50 instances. We chose one of the instances as the experimental standard; thus, the data are divided into two categories. The selected 50 instances are divided into one class, and the other 100 instances are divided into another class. Each instance has four attributes: sepal length, sepal width, petal length, and petal width. In our research, we use the class Iris Versicolour as the output, and thus, it becomes a binary classification. Because of the limitations of the single neuron model, the DE-DMAS can only solve binary classification problems. So we apply the iris dataset as a binary classification problem.

The liver disorders dataset was provided by Richard S. Forsyth in “None known other than what is shown in the PC/BEAGLE User's Guide.” It has been applied in [40, 53]. The dataset has 345 instances. Each instance has six attributes, which include five kinds of blood tests and average daily alcohol consumption. The liver dataset has two classifications: 164 healthy disorders and 181 unhealthy disorders.

The breast cancer data were provided by Dr. William Wolberg in July 1992 [54, 55]. It has been applied in [41]. The 699 instances of these data consist of 458 benign instances and 241 malignant instances. Breast cancer data can be divided into two classes. The breast cancer data include 9 attributes, such as clump thickness, uniformity of cell size and shape, and marginal adhesion.

The glass identification database was provided by B. Herman in September 1987. It has been applied in [56]. The glass data include 163 window glass instances and 51 nonwindow glass instances for a total of 214 instances. The attributes of the glass data include various element contents (Na, Mg, Al, Si, K, Ca, Ba, and Fe) and the refractive index (RI). The instances can be classified by these 9 attributes.

The ACA data indicate whether the applicants are creditworthy. It has been applied in [57, 58]. The credit history of the applicants classifies the data into two classes. These data provide information about 690 applicants. The applicants include 307 people who are creditworthy and 383 people who have no credit. The information that can be considered as the attributes of the ACA dataset consist of 8 categorical records and 6 numerical records.

### 4.2. Optimal Parameter Settings

Three parameters, *F*, CR, and NP, are mentioned in the DE learning algorithm. The number of hidden layers is an important parameter in DE-DMAS, namely, *M*. For different datasets, *M* should be suitably determined. The parameter ranges in DE-DMAS are shown in Table 2.

Typically, we need to experiment with all combinations of parameters to obtain the optimal parameters. However, four parameters exist, and each parameter has three choices. Thus, we should perform 81 (3^4^) different experiments, which will be time-consuming. To ensure the credibility of the experimental results, we should repeat every different experiment 30 times. Because this approach is time-consuming, we should reduce the number of different experiments. Taguchi's method is a kind of method to efficiently obtain the optimal parameters [59, 60]. This method is primarily employed using orthogonal arrays. According to the previously mentioned parameter ranges, four parameter trials containing three datasets are available. Thus, the *L*_9_(3^4^) orthogonal array has been applied in the optimal parameter experiments of the five datasets. The instances of each dataset have been divided into 70% for training and 30% for testing. The orthogonal experiments of each dataset have been repeated 30 times. The epoch of each orthogonal experiment is set to 1000. The orthogonal experimental result of the iris dataset is shown in Table 3. The result of the liver dataset is shown in Table 4. The result of the glass dataset is shown in Table 5. The result of the cancer dataset is shown in Table 6. The result of the ACA dataset is shown in Table 7. The last column displays the average correct rate of 30 test experiments. We obtain the most optimal parameters by a comprehensive analysis of the mean and variance. The bold font indicates the optimal combination of parameters. The optimal parameters for all datasets are shown in Table 8.

### 4.3. Adaptability of Synapses

In order to demonstrate the adaptability of the synaptic layer in DE-DMAS, we carried out a confront analysis. We removed the hyperparameter *k* from the population of DE and set it to 1, 5, and 10, respectively. The five datasets were randomly divided into two parts: 70% for training and 30% for testing. The parameters except *k* were set as the same as these in Table 8. Then, we did 30 independent experiments for them. We recorded all test accuracy results and compared the results in terms of mean and standard deviation, as shown in Table 9. From it, we found that the adaptive *k* which was learned by DE generally performed better than these fixed values. Additionally, the Friedman test [61] gave the statistical analysis results for the accuracies. In this case, the lower the value of the Friedman test, the better the performance. The result of the Friedman test is shown in Table 10. Based on the above results, it is evident that the adaptive synapse is beneficial for DNM.

### 4.4. Comparison with BPNN

In this section, we compared DE-DMAS with the most popular model BPNN. To make the comparison relatively fair, the number of adjusted weights and thresholds (*D*_BPNN_ and *D*_DE−OMAS_ shown in equations (10) and (11), respectively) in both models should be arranged nearly the same because these numbers generally determine the size of the model and the computational complexity although the two models have different architectures:

(10) $$\begin{array}{c} D_{\text{BPNN}}=\left(\text{input}\times \text{hidden}\right)+\left(\text{hidden}\times \text{output}\right)+\text{hidden bias}+\text{output bias}, \end{array}$$

(11) $$\begin{array}{c} D_{\text{DE}-\text{DMAS}}=\left(\text{input}\times \text{hidden}\right)+1. \end{array}$$

However, the larger the number of weights is, the more the occupied computing resources are. In our research, to demonstrate the excellent performance of DE-DMAS for five datasets, the structure of DE-DMAS should be set smaller than that of BPNN. Because the input and output of each dataset are fixed, they are given the same number of weights by adjusting their number of hidden layers. In the previous section, we have configured this parameter (the number of hidden layers) for DE-DMAS. We configure the BPNN with the number of hidden layers according to the above principles. The structures of BPNN and DE-DMAS for the five datasets are shown in Table 11. The learning rate is set to be 0.1 according to the experience.

### 4.5. Comparison with BP-DNM

In order to compare BP-DNM and DE-DMAS fairly, the three common parameters of *k*_soma_, *θ*_soma_, and the number of neurons in hidden layers (*M*) are set to be the same. According to the experience, the learning rate is set to 0.01, and the value of *k* is set to 3. BP-DNM is also a single neuron model with synaptic nonlinearities. It has been proven to have outstanding performance in the liver [40], cancer [41], and ACA [58]. We will show the performs of BP-DNM when it has the same structure as DE-DMAS. We will use multiple objective methods to demonstrate the performances of DE-DMAS and BP-DNM on the five datasets and make a discussion.

## 5. Experimental Result Analysis

The comparison experiment of DE-DMAS vs BPNN and DE-DMAS vs BP-DNM is set up as follows: (1) the instances of the five datasets are divided into 30% for testing and 70% for training randomly, (2) the number of iterations is set to 1000, and (3) all experiments are run using Matlab 2018a.

### 5.1. Convergence Comparison

We use the value of the mean square error (MSE) to represent the degree of convergence. The smaller the value is, the better the convergence is. We calculate the value of MSE after each iteration in the DE-DMAS, BP-DNM, and BPNN training process and record it. We employ the following equation to calculate the value of the MSE:

(12) $$\begin{array}{c} \text{MSE}=\frac{1}{2N}\overset{N}{\underset{i=1}{\sum }}{\left(O_{i}-T_{i}\right)}^{2}, \end{array}$$

where *N* represents the number of training instances and *O*_*i*_ and *T*_*i*_ represent the output and the teacher signal of the *i*-th training instance, respectively.

**Figure 4:**
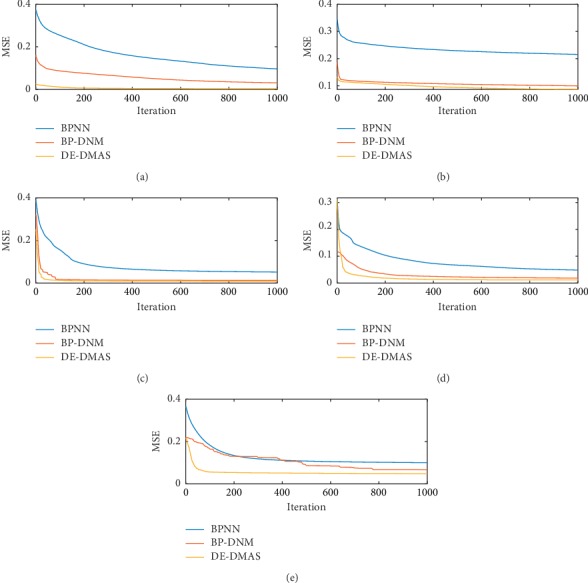
Convergence graphs for the five datasets: (a) iris; (b) liver; (c) cancer; (d) glass; (e) ACA.

We perform 30 training sessions for DE-DMAS, BP-DNM, and BPNN. We randomly select 70% of the instances as the input for each training. We draw two graphs to analyze the convergence effect of DE-DMAS, BP-DNM, and BPNN for the five training datasets. In the first figure, the ordinate represents the mean value of the MSE for 30 training sessions, and the abscissa represents the number of iterations. A total of 1000 MSE values are recorded from the start of initialization for the DE-DMAS, BP-DNM, and BPNN. We can evaluate the speed of convergence by the degree of the curve drop. In Figure 4, we note that curve of DE-DMAS is falling faster than the comparators. These figures show that DE-DMAS has an advantage in convergence speed.

**Figure 5:**
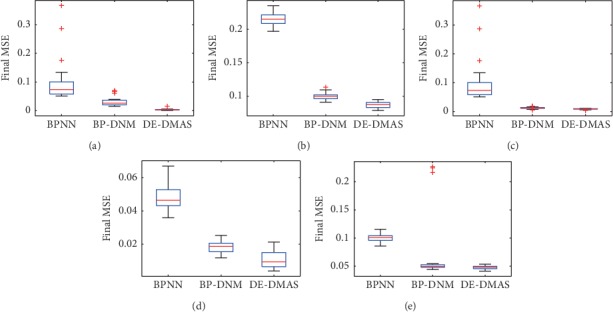
Box-and-whisker plots for the final MSE: (a) iris; (b) liver; (c) cancer; (d) glass; (e) ACA.

We record the value of MSE in the final iteration for 30 times in the training sessions. We use a box-and-whisker plot [62] to represent the value of the MSE, as shown in Figure 5. In this figure, the ordinate represents the value of the MSE. The horizontal line from the top to the bottom of each box represents the maximum, 3/4 median, median, 1/4 median, and minimum. The 1/4 median, median, and 3/4 median represent the value of MSE at 25%, 50%, and 75%, respectively, after sorting. The +sign represents an outlier, which is a value that exceeds twice the standard deviation. The lines corresponding to the maximum, 3/4 median line, median, 1/4 median, and minimum for DE-DMAS are below those of BP-DNM and BPNN for the five datasets. Many outliers exist in the boxes of BPNN and BP-DNM. The outliers above the maximum represent falling into a local minimum during training. On the contrary, there is no outlier above the maximum in the boxes of DE-DMAS. The results show that the convergence effect of DE-DMAS is better than that of BP-DNM and BPNN.

### 5.2. Accuracy Comparison

We compare DE-DMAS, BP-DNM, and BPNN in terms of the test accuracy, sensitivity, specificity, and receiver operating characteristic (ROC) curve [63], which is a method for objectively analyzing the performance of classifiers. To draw the ROC curve, we collected the output (*O*) of the five datasets tested by DE-DMAS, BP-DNM, and BPNN. *T* represents the corresponding teacher signals. We convert the output *O* from a real number to an integer of 0 or 1. For a two-category problem, the instances are divided into positive and negative classes. The actual classification has four situations:

1. If an instance is in the positive class and is predicted to be in the positive class, then it is a true classification (true positive (TP))
2. If an instance is in the positive class but is predicted to be in the negative class, then it is a false-negative classification (false negative (FN))
3. If an instance is in the negative class but is predicted to be in the positive class, then it is a false-positive classification (false positive (FP))
4. If an instance is in the negative class and is predicted to be in the negative class, then it is a true-negative classification (true negative (TN))

The true-positive rate (TPR), which represents the proportion of actual positive instances in the positive class predicted by the classifier to all positive instances, equals the sensitivity. The false-positive rate (FPR), which represents the proportion of actual negative instances in the positive class predicted by the classifier to all negative instances, equals 1-specificity. The ROC curve is drawn with the FPR (1 − specificity) as the *x*-axis and the TPR (sensitivity) as the *y*-axis. The AUC is the area under the ROC curve. The value of AUC is between 0.0 and 1.0 since the ROC curve is drawn in an square area. The greater the values of the sensitivity, specificity, and AUC are, the better the performance of the classifier is. These terms are defined in Table 12. We calculate the accuracy, sensitivity, specificity, and AUC based on these terms using the following equations:

(13) $$\begin{array}{c} \text{accuracy}=\frac{\text{TP}+\text{TN}}{\text{TP}+\text{FN}+\text{TN}+\text{FP}},\\ \text{sensitivity}=\frac{\text{TP}}{\left(\text{TP}+\text{FN}\right)},\\ \text{specificity}=\frac{\text{TN}}{\left(\text{TN}+\text{FP}\right)},\\ \text{AUC}\left(\%\right)=\frac{1}{2}\left(\frac{\text{TP}}{\text{TP}+\text{FN}}+\frac{\text{TN}}{\text{TN}+\text{FP}}\right)\times 100. \end{array}$$

**Figure 6:**
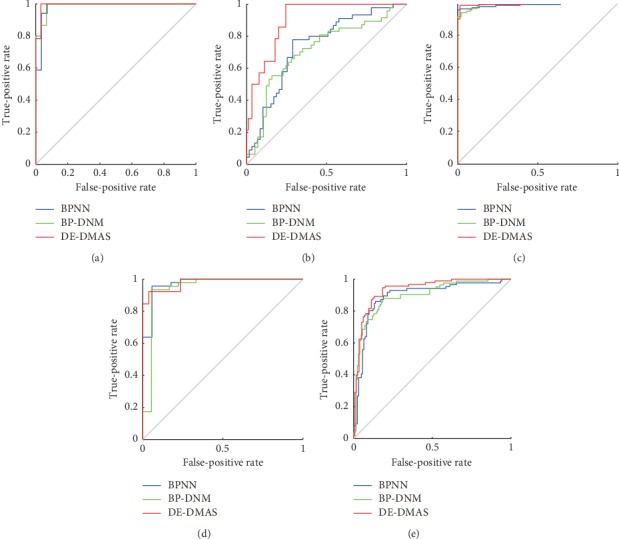
ROC analysis for the five datasets: (a) iris ROC; (b) liver ROC; (c) cancer ROC; (d) glass ROC; (e) ACA ROC.

We plot the ROC curves of the five datasets to compare DE-DMAS with BP-DNM and BPNN, as shown in Figure 6. The DE-DMAS curves are above the BP-DNM and BPNN curves. The sensitivity, specificity, and AUC, which can be determined from the numerical values, are shown in Table 13. The test accuracy is the average of 30 experiments, which we represent by the mean and variance in Table 13. DE-DMAS exhibits higher values than BP-DNM and BPNN for these four assessment levels. All test results prove the superiority of DE-DMAS.

### 5.3. Cross-Validation

In order to facilitate the comparison performance, four different experimental train-to-test ratios were adopted and the four multifold cross-validation (*K* × CV) methods include tenfold CV (90–10%, ×10), fivefold CV (80–20%, ×5), fourfold CV (75–25%, ×4), and twofold CV(50–50%, ×2). Here, the train-to-test ratio represents the ratio between sample size for training and testing. With *K* × CV (*K* = 2, 4, 5, 10), the whole dataset is randomly divided into *K* and mutually exclusive subsets with approximately equal sample size. In *K* × CV, the method is utilized on the training subsets, and the testing error is measured on the testing subset. The procedure is repeated for a total of *K* trials, each time using a different subset for testing. The performance of the model is evaluated by the mean of the squared error through testing over all trails of the experiment. Compared with the single-fold validation method, *K* × CV has an advantage of minimizing the correlation deviation of random sampling of training samples, but its disadvantage lies in that it may need too much computation since the model has to be trained *K* times. We select four kinds of BPNN with different learning rates and number (No.) of branch as Table 14, namely, BPNN1, BPNN2, BPNN3, and BPNN4. BP-DNM and DE-DMAS used the above four types of training-to-test ratios. Five datasets were applied to each type of training-to-test ratios for each model, and 30 independent experiments were performed. Finally, we compared the mean and standard deviation of their test accuracy results.  Table 15 shows the cross-validation results of the iris dataset.  Table 16 shows the cross-validation results of the liver dataset.  Table 17 shows the cross-validation results of the cancer dataset.  Table 18 shows the cross-validation results of the glass dataset.  Table 19 shows the cross-validation results of the ACA dataset. Bold fonts in these tables indicate the top two according to the standard deviation and the mean. DE-DMAS only in the CV5 of the cancer dataset is not bold font. So we can conclude that DE-DMAS is excellent.

### 5.4. Simplified Model

**Figure 7:**
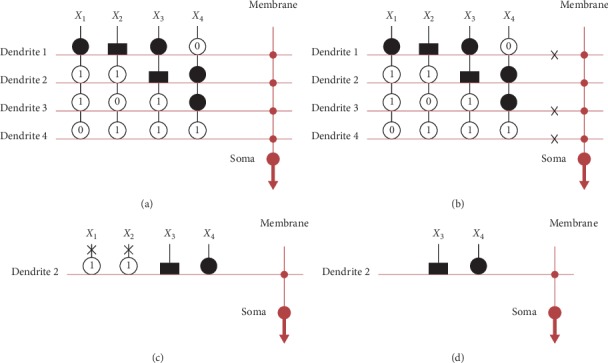
Structure simplification process for the iris dataset.

As previously mentioned, we remove the useless dendrites and synapses by the pruning function. The entire simplification process for the iris dataset is shown in Figure 7. First, initialize the structure of neurons with four dendrites, as shown in Figure 7(a). Synapses on these dendrites receive the inputs *X*_1_, *X*_2_, *X*_3_, and *X*_4_. The synapses are activated and converted to the direct-connecting state (●), opposite-connecting state (^▂^), constant-1 state(①), or constant-0 state (⓪) after learning. Second, remove all useless dendrites by the dendrite pruning function; that is, if at least one synapse on a dendrite is in the constant-0 state, remove the dendrites. In Figure 7(b), we denote removed dendrite 1, dendrite 3, and dendrite 4 with the symbol ✖. After dendrite pruning, only dendrite 2 remains, as shown in Figure 7(c). Then, remove all unnecessary synapses by the synapse pruning function; that is, remove the synapses with the constant-1 state by the symbol ✖ in the Figure 7(c).  Figure 7(d) shows the simplified structure for the iris dataset. This structure is simplified from 4 layers of dendrites and 4 inputs to only dendrite 2 and the 2 inputs *X*_3_ and *X*_4_.

**Figure 8:**
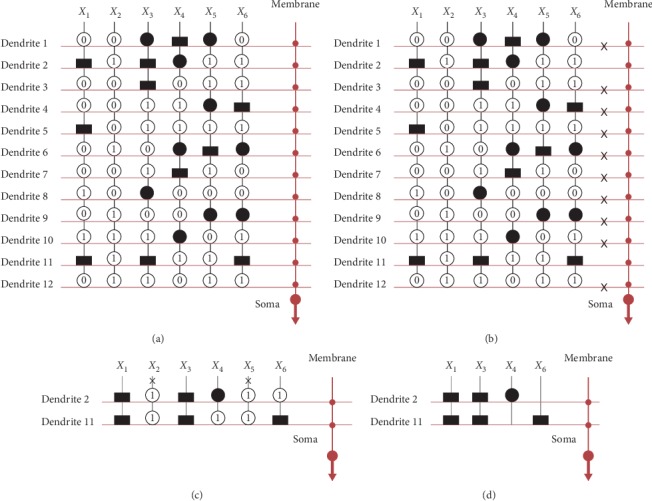
Structure simplification process for the liver dataset.

Liver dataset has 12 layers of dendrites and 6 inputs, as shown in Figure 8(a). In Figure 8(b), we denote removed (dendrites 1, 3, 4, 5, 6, 7, 8, 9, 10, and 12) with the symbol ✖. After dendrite pruning, dendrites 2 and 11 remain, as shown in Figure 8(c). After synapse pruning, *X*_1_, *X*_3_, *X*_4_, and *X*_6_ have been remained, and the others have been removed by the symbol ✖.  Figure 8(d) shows the simplified structure for the liver dataset. This structure is simplified from 12 layers of dendrites and 6 inputs to only dendrites 2 and 11 and the 4 inputs *X*_1_, *X*_3_, *X*_4_, and *X*_6_.

**Figure 9:**
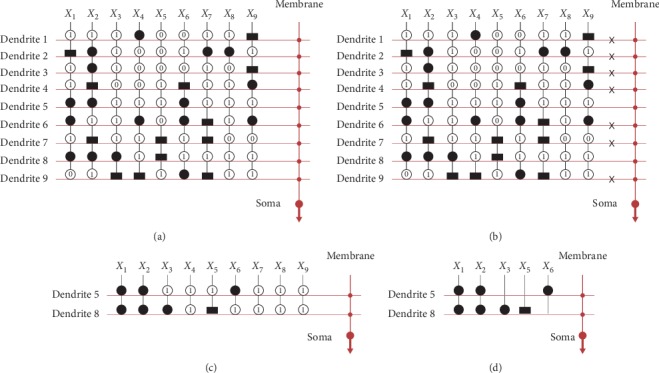
Structure simplification process for the cancer dataset.

Cancer dataset has 9 layers of dendrites and 9 inputs, as shown in Figure 9(a). In Figure 9(b), we denote removed (dendrites 1, 2, 3, 4, 6, 7, and 9) with the symbol ✖. After dendrite pruning, dendrites 5 and 8 remain, as shown in Figure 9(c). After synapse pruning, *X*_1_, *X*_2_, *X*_3_, *X*_5_, and *X*_6_ have been remained, and the others have removed by the symbol ✖.  Figure 9(d) shows the simplified structure for the cancer dataset. This structure is simplified from 9 layers of dendrites and 9 inputs to only dendrites 5 and 8 and the 5 inputs *X*_1_, *X*_2_, *X*_3_, *X*_5_, and *X*_6_.

**Figure 10:**
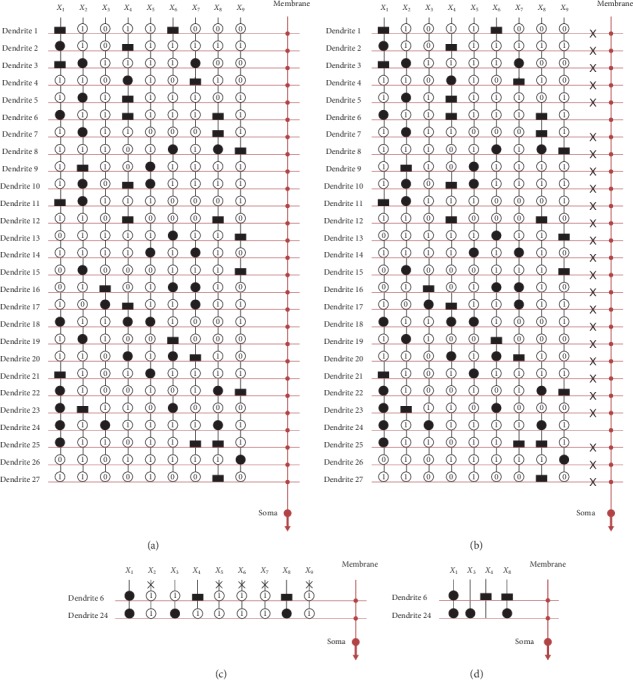
Structure simplification process for the glass dataset.

Glass dataset has 27 layers of dendrites and 9 inputs, as shown in Figure 10(a). In Figure 10(b), we denote removed (dendrites 1, 2, 3, 4, 5, 7, 8, 9, 10, 11, 12, 13, 14, 15, 16, 17, 18, 19, 20, 21, 22, 23, 25, 26, and 27) with the symbol ✖. After dendrite pruning, dendrites 6 and 24 remain, as shown in Figure 10(c). After synapse pruning, *X*_1_, *X*_3_, *X*_4_, and *X*_8_ have been remained, and the others have been removed by the symbol ✖.  Figure 10(d) shows the simplified structure for the liver dataset. This structure is simplified from 12 layers of dendrites and 6 inputs to only dendrites 6 and 24 and the 4 inputs *X*_1_, *X*_3_, *X*_4_, and *X*_8_.

**Figure 11:**
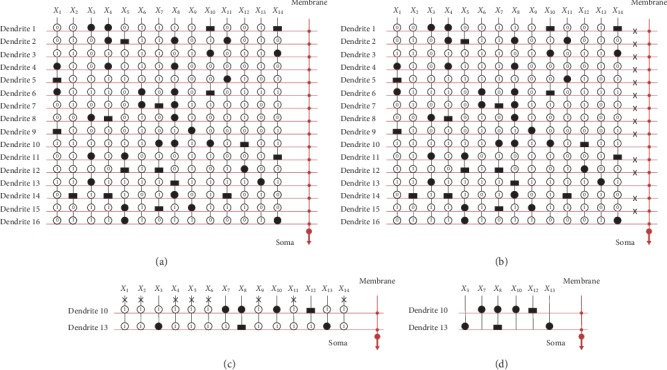
Structure simplification process for the ACA dataset.

ACA dataset has 16 layers of dendrites and 14 inputs, as shown in Figure 11(a). In Figure 11(b), we denote removed (dendrites 1, 2, 3, 4, 6, 7, 8, 9, 11, 12, 14, 15, and 16) with the symbol ✖. After dendrite pruning, dendrites 10 and 13 remain, as shown in Figure 11(c). After synapse pruning, *X*_3_, *X*_7_, *X*_8_, *X*_10_, *X*_12_, and *X*_13_ have remained, and the others have been removed by the symbol ✖.  Figure 11(d) shows the simplified structure for the liver dataset. This structure is simplified from 16 layers of dendrites and 6 inputs to only dendrites 10 and 13 and the 6 inputs *X*_3_, *X*_7_, *X*_8_, *X*_10_, *X*_12_, and *X*_13_.

**Figure 12:**
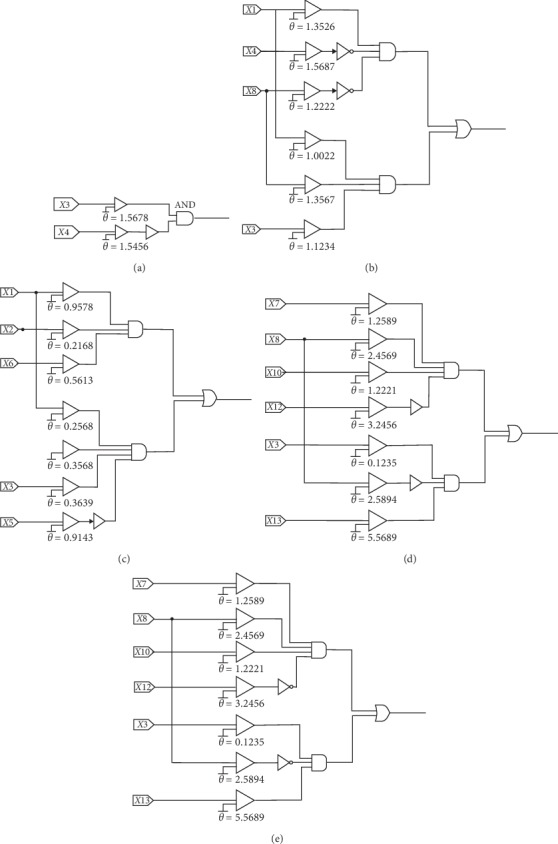
Logic circuits obtained by the proposed method for five datasets: (a) iris; (b) liver; (c) cancer; (d) glass; (e) ACA.

As shown in these figures, we have obtained the final simplified models of the five datasets. After simplifying the models, the structures of the models have been reduced by more than 90%, which indicates that we can use simpler logic to solve the real problem. The problem used to be solved with more than hundreds of logic components but can now be solved by only a few dozen simple logic components, such as comparators, AND gates, OR gates, and NOT gates. This change substantially reduces the labor and time costs. Logic circuits of the five datasets have been drawn in Figure 12. The value of *θ* in the comparator in the figure is the value of *θ* in the corresponding synapse after training. The standardized input was calculated by these logic components to get the expected output as 0 or 1.

## 6. Conclusion

To improve the calculation ability of the dendritic neuron model (DNM), a dendritic neuron model with adaptive synapses trained by differential evolution algorithm (DE-DMAS) is proposed, which shows enhanced performance in the simulation based on UCI datasets. A comparison with the classic BPNN and BP-DNM is carried out in terms of the test accuracy, sensitivity, specificity, and ROC and cross-validation. DE-DMAS shows its superiority in all the results, and DE-DMAS as a single neuron model is found to substantially outperform BPNN and BP-DNM.

DMAS has further access to the real biological neuron with a self-pruning ability. This function can eliminate branches from the dendrite morphology depending on the continuum values. It hence reduces the computational load by evolving and simplifying the dendritic structure without affecting the computational result. Simplified dendritic structure can be implemented in the logic circuit with comparator, OR gate, AND gate, and NOT gate. It makes it possible to solve real problems with less cost.

To highlight the contribution of this work, a self-adaptive synapse is for the first time proposed in the paper. Its utility is proved by the Friedman test as summarized in Section 4.3. The ability of adaptive synapse not only has stronger robustness but also reduces a parameter in DNM and improves the performance of DNM.

The dendrite plays a pivotal role in the computing process. A single DE-DMAS neuron model can only deal with dichotomies problems (i.e., binary classification problems), which is its main limitation. But all the current neural networks are made up of multiple single neuron models which can only deal with dichotomies. This paper aims at proposing the DE-DMAS model instead of the network structure, so it is only a single one. Nevertheless, it is worth pointing out that variants of DE-DMAS can be developed for solving multiclass classification problems. For example, by using softmax function (together with the cross entropy), the multiclass classification problem can be approximately transformed into dichotomies problems, and the one-hot-encoding strategy based on several DE-DMAS neuron models can be used to calculate the information entropy. The reason why we choose multiple datasets that can be classified into two categories for experiments is that we want to more intuitively reflect the ability of a single neuron model, rather than the network of several ones. Further research will focus on DMAS's adjustment to make it adaptive to the deep learning structure. We also believe that this model has considerable potential in fields of electronic design, such as VLSI and biomedical science.

## Figures and Tables

**Algorithm 1:**
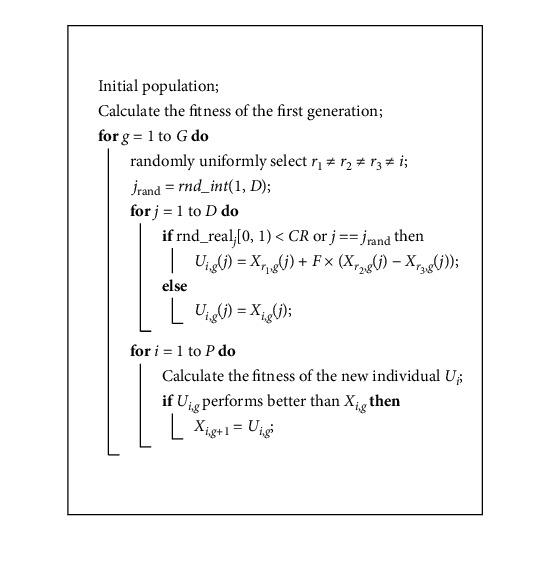
Differential evolution algorithm for DNM.

**Table 1:** Dataset introduction.

Dataset name	No. of instances	No. of input attributes	No. of class 1 instances	No. of class 2 instances
Iris	150	4	50	100
Liver	345	6	164	181
Cancer	699	9	458	241
Glass	214	9	51	163
ACA	690	14	307	383

**Table 2:** Parameter ranges in DE-DMAS.

Dataset	NP	CR	*F*	*M*
Iris	10, 30, 60	0.3, 0.6, 0.9	0.3, 0.6, 0.9	4, 8, 12
Liver	10, 30, 60	0.3, 0.6, 0.9	0.3, 0.6, 0.9	6, 12, 18
Cancer	10, 30, 60	0.3, 0.6, 0.9	0.3, 0.6, 0.9	9, 18, 27
Glass	10, 30, 60	0.3, 0.6, 0.9	0.3, 0.6, 0.9	9, 18, 27
ACA	10, 30, 60	0.3, 0.6, 0.9	0.3, 0.6, 0.9	16, 32, 48

**Table 3:** Orthogonal array for parameters of the iris dataset.

Experimental runs	Parameters (levels)				Accuracy (%)
	Branch (3)	*F* (3)	CR (3)	NP (3)	
1	4	0.3	0.3	10	92.51 ± 4.61
2	8	0.3	0.6	30	94.49 ± 5.46
3	12	0.3	0.9	60	94.96 ± 2.47
4	12	0.6	0.3	30	94.20 ± 4.61
5	4	0.6	0.6	10	**96.74** ± **2.78**
6	8	0.6	0.9	60	93.62 ± 5.32
7	8	0.9	0.3	60	94.20 ± 4.47
8	12	0.9	0.6	10	95.50 ± 4.90
9	4	0.9	0.9	30	94.00 ± 2.74

**Table 4:** Orthogonal array for parameters of the liver dataset.

Experimental runs	Parameters (levels)				Accuracy (%)
	Branch (3)	*F* (3)	CR (3)	NP (3)	
1	6	0.3	0.3	10	70.60 ± 4.81
2	12	0.3	0.6	30	**73.59** ± **6.66**
3	18	0.3	0.9	60	72.17 ± 3.86
4	18	0.6	0.3	30	66.92 ± 6.80
5	6	0.6	0.6	10	71.85 ± 4.75
6	12	0.6	0.9	60	68.33 ± 6.91
7	12	0.9	0.3	60	66.28 ± 7.36
8	18	0.9	0.6	10	68.58 ± 7.74
9	6	0.9	0.9	30	69.61 ± 4.88

**Table 5:** Orthogonal array for parameters of the glass dataset.

Experimental runs	Parameters (levels)				Accuracy (%)
	Branch (3)	*F* (3)	CR (3)	NP (3)	
1	9	0.3	0.3	10	93.75 ± 2.65
2	18	0.3	0.6	30	94.37 ± 4.13
3	27	0.3	0.9	60	**94.42** ± **2.51**
4	27	0.6	0.3	30	94.06 ± 4.44
5	9	0.6	0.6	10	93.12 ± 4.29
6	18	0.6	0.9	60	93.02 ± 4.07
7	18	0.9	0.3	60	93.54 ± 3.66
8	27	0.9	0.6	10	92.81 ± 4.03
9	9	0.9	0.9	30	94.01 ± 2.66

**Table 6:** Orthogonal array for parameters of the cancer dataset.

Experimental runs	Parameters (levels)				Accuracy (%)
	Branch (3)	*F* (3)	CR (3)	NP (3)	
1	9	0.3	0.3	10	**96.12** ± **1.17**
2	18	0.3	0.6	30	96.12 ± 1.65
3	27	0.3	0.9	60	95.80 ± 1.26
4	27	0.6	0.3	30	95.39 ± 1.97
5	9	0.6	0.6	10	96.09 ± 1.64
6	18	0.6	0.9	60	96.19 ± 1.90
7	18	0.9	0.3	60	96.20 ± 2.00
8	27	0.9	0.6	10	95.81 ± 1.55
9	9	0.9	0.9	30	95.84 ± 1.31

**Table 7:** Orthogonal array for parameters of the ACA dataset.

Experimental runs	Parameters (levels)				Accuracy (%)
	Branch (3)	*F* (3)	CR (3)	NP (3)	
1	16	0.3	0.3	10	84.41 ± 5.00
2	32	0.3	0.6	30	85.81 ± 2.41
3	48	0.3	0.9	60	85.28 ± 2.28
4	48	0.6	0.3	30	85.18 ± 1.67
5	16	0.6	0.6	10	85.58 ± 2.14
6	32	0.6	0.9	60	85.70 ± 1.64
7	32	0.9	0.3	60	84.89 ± 2.26
8	48	0.9	0.6	10	84.41 ± 5.73
9	16	0.9	0.9	30	**86.18** ± **1.98**

**Table 8:** Parameters for DE-DMAS

Dataset	NP	CR	*F*	*M*
Iris	0.6	0.6	10	4
Liver	0.3	0.6	30	12
Cancer	0.3	0.3	10	9
Glass	0.3	0.9	60	27
ACA	0.9	0.9	30	16

**Table 9:** Demonstrate on the adaptability of synapse in terms of test accuracy.

Dataset	*k* = 1	*k* = 5	*k* = 10	Adaptive
Iris	80.81 ± 6.66	94.15 ± 2.83	95.11 ± 2.50	96.74 ± 2.78
Liver	69.39 ± 3.57	69.81 ± 4.12	69.39 ± 5.56	73.59 ± 6.66
Cancer	92.76 ± 3.35	92.97 ± 3.38	93.59 ± 2.11	96.12 ± 1.17
Glass	91.04 ± 2.84	93.33 ± 3.23	85.48 ± 2.02	94.42 ± 2.51
ACA	85.44 ± 2.99	85.56 ± 1.70	85.86 ± 2.53	86.18 ± 1.98

**Table 10:** Demonstrate on the adaptability of synapse by the Friedman test.

Dataset	*k* = 1	*k* = 5	*k* = 10	Adaptive
Iris	4	2.33	2	1.67
Liver	2.77	2.73	2.55	1.95
Cancer	3.05	2.95	2.73	1.27
Glass	2.55	1.98	3.93	1.53
ACA	2.48	2.73	2.52	2.27

**Table 11:** Structures of DE-DMAS and BPNN for the five datasets.

Dataset	Method	No. of inputs	No. of branches	No. of outputs	No. of adjusted weights
Iris	DE-DMAS	4	4	1	33
	BPNN	4	28	1	169

Liver	DE-DMAS	6	12	1	145
	BPNN	6	18	1	145

Cancer	DE-DMAS	9	9	1	163
	BPNN	9	18	1	199

Glass	DE-DMAS	9	27	1	487
	BPNN	27	45	1	496

ACA	DE-DMAS	14	16	1	449
	BPNN	14	30	1	481

**Table 12:** Description of terms.

Teacher signal	Real output		Row total
	Positive (1)	Negative (0)	
Positive (1)	TP	FN	TP + FN
Negative (0)	FP	TN	FP + TN
Column total	TP + F	FN + TN	N = TP + TN + FP + FN

**Table 13:** Rate results for the five datasets.

Dataset	Method	Test accuracy	Sensitivity	Specificity	AUC
Iris	DE-DMAS	96.74 ± 2.78	100	96.88	98.44
	BP-DNM	91.78 ± 5.87	91.16	86.67	95.65
	BPNN	85.93 ± 10.89	90.00	91.43	90.71

Liver	DE-DMAS	73.59 ± 6.66	66.67	83.87	75.27
	BP-DNM	68.62 ± 5.24	52.50	.79.69	66.09
	BPNN	59.94 ± 6.33	57.89	72.73	65.31

Cancer	DE-DMAS	96.12 ± 1.17	96.45	98.55	97.50
	BP-DNM	96.33 ± 1.43	97.04	94.67	95.85
	BPNN	93.76 ± 11.06	95.89	96.88	96.38

Glass	DE-DMAS	94.42 ± 2.51	98.57	92.85	96..67
	BP-DNM	91.87 ± 3.88	84.62	96.08	90.35
	BPNN	92.50 ± 3.34	97.92	81.25	89.58

ACA	DE-DMAS	86.18 ± 1.98	88.04	86.09	87.07
	BP-DNM	83.66 ± 9.26	85.54	81.45	83.50
	BPNN	85.57 ± 2.28	87.10	78.07	82.58

**Table 14:** No. of models.

No. of BPNN	Learning rate	No. of branch
BPNN1	0.1	30
BPNN2	0.08	60
BPNN3	0.08	30
BPNN4	0.06	60

**Table 15:** Cross-validation for the iris.

Model	CV10	CV5	CV4	CV2
DE-DMAS	**94.44** ± **5.59**	**94.56** ± **5.90**	**95.00** ± **3.61**	**93.38** ± **5.09**
BP-DNM	93.33 ± 10.79	93.22 ± 6.64	91.93 ± 4.98	86.80 ± 9.16
BPNN1	90.67 ± 7.50	91.42 ± 5.62	89.06 ± 9.41	91.29 ± 7.182
BPNN2	**93.55** ± **10.72**	**93.35** ± **7.92**	**92.80** ± **7.82**	**91.82** ± **7.97**
BPNN3	91.10 ± 9.85	88.77 ± 9.20	90.27 ± 9.89	86.71 ± 13.43
BPNN4	90.44 ± 8.69	89.89 ± 9.07	88.80 ± 9.59	89.07 ± 9.72

**Table 16:** Cross-validation for the liver.

Model	CV10	CV5	CV4	CV2
DE-DMAS	**70.20** ± **7.98**	**71.93** ± **4.79**	**71.40** ± **0.40**	**70.12** ± **3.10**
BP-DNM	58.82 ± 7.93	**69.42** ± **5.68**	**68.88** ± **5.57**	**66.44** ± **4.81**
BPNN1	58.76 ± 9.45	59.18 ± 5.72	59.43 ± 7.34	60.44 ± 4.30
BPNN2	**63.14** ± **8.20**	58.72 ± 6.11	61.47 ± 6.72	58.83 ± 5.05
BPNN3	58.00 ± 8.33	58.55 ± 5.80	61.47 ± 6.72	59.37 ± 8.50
BPNN4	60.19 ± 8.94	61.11 ± 6.09	60.92 ± 8.45	60.71 ± 5.56

**Table 17:** Cross-validation for cancer.

Model	CV10	CV5	CV4	CV2
DE-DMAS	**96.38** ± **2.36**	96.05 ± 1.74	**96.11** ± **1.12**	**95.90** ± **0.70**
BP-DNM	95.71 ± 2.17	96.02 ± 1.84	95.75 ± 1.64	95.70 ± 1.06
BPNN1	91.48 ± 11.65	92.26 ± 11.80	**96.29** ± **1.67**	94.29 ± 1.08
BPNN2	**96.57** ± **2.51**	**96.28** ± **1.54**	94.33 ± 1.52	93.71 ± 2.44
BPNN3	93.45 ± 12.00	95.52 ± 1.45	95.73 ± 1.35	93.53 ± 11.54
BPNN4	94.05 ± 12.53	**96.30** ± **1.34**	96.10 ± 1.75	**96.07** ± **1.04**

**Table 18:** Cross-validation for glass.

Model	CV10	CV5	CV4	CV2
DE-DMAS	**95.24** ± **3.75**	**96.05** ± **1.74**	**96.01** ± **1.36**	**95.70** ± **0.70**
BP-DNM1	**92.53** ± **4.95**	92.40 ± 3.23	**91.58** ± **3.16**	91.12 ± 2.77
BPNN1	91.59 ± 5.55	90.39 ± 4.44	91.05 ± 3.19	91.53 ± 2.22
BPNN2	91.90 ± 4.36	**92.56** ± **4.12**	90.99 ± 4.12	90.31 ± 5.24
BPNN3	91.75 ± 4.83	89.61 ± 4.56	91.42 ± 4.28	89.50 ± 3.85
BPNN4	90.00 ± 6.77	92.02 ± 4.08	90.00 ± 5.36	**91.78** ± **3.72**

**Table 19:** Cross-validation for ACA.

Model	CV10	CV5	CV4	CV2
DE-DMAS	**84.98** ± **3.95**	**86.79** ± **2.70**	**85.59** ± **2.81**	**85.78** ± **1.39**
BP-DNM	83.09 ± 8.52	82.00 ± 10.62	83.06 ± 10.51	82.07 ± 10.36
BPNN1	**85.94** ± **4.34**	85.20 ± 2.66	**85.04** ± **2.63**	85.39 ± 1.61
BPNN2	84.12 ± 3.97	85.11 ± 2.60	83.51 ± 3.41	**86.13** ± **1.28**
BPNN3	83.51 ± 4.64	85.48 ± 2.79	84.00 ± 3.49	85.35 ± 1.18
BPNN4	84.16 ± 4.48	**86.11** ± **2.15**	83.51 ± 33.30	85.09 ± 1.55

## Data Availability

The classification dataset could be downloaded freely at https://archive.ics.uci.edu/ml/index.php.
